# The Mitochondrial Pentatricopeptide Repeat Protein PPR18 Is Required for the *cis*-Splicing of *nad4* Intron 1 and Essential to Seed Development in Maize

**DOI:** 10.3390/ijms21114047

**Published:** 2020-06-05

**Authors:** Rui Liu, Shi-Kai Cao, Aqib Sayyed, Chunhui Xu, Feng Sun, Xiaomin Wang, Bao-Cai Tan

**Affiliations:** 1 Key Laboratory of Plant Development and Environment Adaptation Biology, Ministry of Education, School of Life Sciences, Shandong University, Qingdao 266237, China; liuxiaoshuang_6@163.com (R.L.); caoshk5233@163.com (S.-K.C.); aqib.sayyed@yahoo.com (A.S.); chunhuixu@sdu.edu.cn (C.X.); epusun@sdu.edu.cn (F.S.); 2 Key Laboratory of Cell Activities and Stress Adaptations, Ministry of Education, School of Life Sciences, Lanzhou University, Lanzhou 730000, China; wangxiaomin@lzu.edu.cn

**Keywords:** PPR protein, mitochondrial complex I, *nad4*, RNA splicing, seed development, maize

## Abstract

Pentatricopeptide repeat (PPR) protein comprises a large family, participating in various aspects of organellar RNA metabolism in land plants. There are approximately 600 PPR proteins in maize, but the functions of many PPR proteins remain unknown. In this study, we defined the function of PPR18 in the *cis*-splicing of *nad4* intron 1 in mitochondria and seed development in maize. Loss function of *PPR18* seriously impairs embryo and endosperm development, resulting in the *empty pericarp* (*emp*) phenotype in maize. *PPR18* encodes a mitochondrion-targeted P-type PPR protein with 18 PPR motifs. Transcripts analysis indicated that the splicing of *nad4* intron 1 is impaired in the *ppr18* mutant, resulting in the absence of *nad4* transcript, leading to severely reduced assembly and activity of mitochondrial complex I and dramatically reduced respiration rate. These results demonstrate that PPR18 is required for the *cis*-splicing of *nad4* intron 1 in mitochondria, and critical to complex I assembly and seed development in maize.

## 1. Introduction

Mitochondria are originated from α-proteobacteria ancestors via endosymbiosis. During evolution, the majority of the bacterial ancestral genes from mitochondrial genome have been lost or transferred to host nucleus [1]. In angiosperms, mitochondrial genomes contain up to 60 genes, which are involved in biogenesis of respiratory complex subunits, ribosomal proteins, ribosomal RNAs (rRNAs), and transfer RNAs (tRNAs) [2,3]. Maize mitochondrial genome contains 58 identified genes that encode 22 proteins of the electron transport chain, 9 ribosomal proteins, a maturase (MatR), a transporter protein (MttB), 3 ribosomal RNAs (5S, 18S, and 26S), and 21 tRNAs [4].

Mature mitochondrial transcripts undergo extensive post-transcriptional processing events, among which the most reported are RNA editing and RNA splicing [5,6,7]. In flowering plants, RNA editing usually alters cytidine to uridine through a deamination reaction in mitochondria and plastids and RNA splicing is a processing event in which noncoding segments (intron) of precursor RNA are removed and coding sequences are joined. Based on the distinctive structures, introns are divided into two families, group I and group II [4,8]. In flowering plants, most of organellar transcripts contain group II introns with conserved secondary structure, consisting of six domains extending from a central hub [9]. In bacteria, the splicing of a group II intron is self-facilitated by its cognate maturase encoded in the intron domain IV [10], but in plants, nearly all introns lost the maturase gene with only one intron maturase gene (*matK*) remains in the plastid genome and one *matR* gene in the mitochondrial genome [11,12]. Instead, four maturase genes (*nMat1* to *4*) are found in the nuclear genome in *Arabidopsis* [13,14,15]. Besides, numerous additional nucleus-encoded splicing co-factors have been reported to be involved in the splicing of organellar introns, such as the chloroplast RNA splicing and ribosome maturation (CRM) domain-containing proteins [16,17], RNA helicase [18,19], mitochondrial transcription termination factors (mTERF) [20,21], plant organellar RNA recognition (PORR) domain proteins [22,23], regulator of chromosome condensation (RCC1) domain proteins [24], and the pentatricopeptide repeat (PPR) proteins [5,25,26].

PPR proteins are a large family of RNA binding proteins, with more than 400 members in angiosperms [5,27]. PPR proteins contain multiple 35-amino-acid tandem repeats and each repeat forms a helix-loop-helix structure. Based on domain constitution, PPR proteins are divided into PLS (repeat P–L–S motif)-class proteins and P-class proteins [27]. The PLS-subclass PPR proteins contain characteristic triplets of P, L, and S motifs with additional E, E+, DYW, or other domains at the C-terminus, whereas the P-subclass PPR proteins contain arrays of only P motifs [5]. The PLS-class PPR proteins are implicated in the C-to-U RNA editing that in most cases is to restore the evolutionary conserved amino acids [28]. Functions of the P-subclass PPR proteins are diverse, which includes RNA cleavage, RNA splicing, RNA stabilization and maturation, and translation initiation [5]. Most PPR proteins are localized in mitochondria or chloroplasts. They bind RNA in a sequence specific manner that one PPR motif binds to one nucleotide of the target RNA. The recognition nucleotides were determined by the different combinations of the amino acid residues at position 5th and 35th of each PPR repeat, which is known as the PPR codes [29,30,31]. In plant mitochondria, most of group II introns are present in genes that code for subunits of mitochondrial complex I. In maize mitochondria, out of 22 identified group II introns, 19 resides in *nad1*, *nad2*, *nad4*, *nad5*, and *nad7* transcripts, while 3 in *rps3*, *cox2*, and *ccmFc* transcripts [32,33]. Accurate splicing of these group II introns is critical to mitochondrial function and biogenesis, which is important for plant growth and development. For instance, EMP11 and DEK2 are involved in the splicing of *nad1* introns, and the loss of function mutation of *Emp11* and *Dek2* affects the assembly of complex I with severely arrested embryo and endosperm development [34,35]. EMP10, EMP12, EMP16, DEK37, and PPR20 are responsible for the splicing of *nad2* introns in maize. These mutations result in a loss of mitochondrial complex I assembly and activity, impairing the mitochondrial function and embryogenesis and endosperm development [25,36,37,38,39].

In this study, we characterized a maize seed mutant *ppr18*, which exhibits arrested embryo and endosperm development phenotype. *PPR18* encodes a mitochondrion-targeted P-type PPR protein with 18 PPR motifs. The loss of *PPR18* function leads to the splicing deficiency of *nad4* intron 1, severely reduced assembly and activity of mitochondrial complex I, resulting in the impairment of mitochondrial function and seed development in maize.

## 2. Results

### 2.1. PPR18 Is a Mitochondrion-Localized P-Type PPR Protein

**Figure 1:**
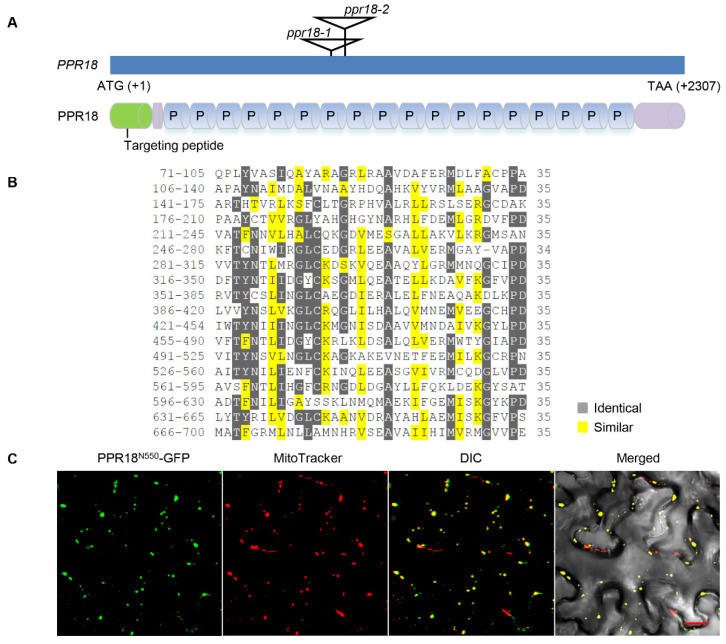
PPR18 is a mitochondrion-localized P-type pentatricopeptide repeat (PPR) protein. (**A**) Schematic illustrating the genomic structure and protein structure of *PPR18*. The locations of the *Mu* insertions are marked with triangles in two independent alleles. P, P-type PPR motif. (**B**) Alignment analysis of 18 PPR motifs in PPR18 protein. Identical amino acids are highlighted in dark gray and similar ones in yellow. (**C**) Localization of PPR18^N550^-GFP in tobacco mesophyll cells. Green fluorescence, red fluorescence, and yellow fluorescence (merge) show green fluorescent protein (GFP) fluorescence, MitoTracker stained mitochondria, and localization, respectively. DIC, differential interference contrast. Scale bar, 20 μm.

*PPR18* (GRMZM2G438456) is an intronless gene, encoding an 85 kDa protein with 768 amino acid residues (Figure 1A). Motif prediction analysis by algorithm TPRpred (http://tprpred.tebingen.mpg.de/tprpred) revealed that PPR18 contained 18 tandemly repeated PPR motifs without any other domains, suggesting that PPR18 is a canonical P-type PPR protein (Figure 1A,B). A phylogenetic analysis based on the maize PPR18 and its homologous proteins revealed extensive conservation in the sequences in both monocots and dicots (Figure S1). Most of PPRs are localized in organelles, either chloroplasts or/and mitochondria, except GRP23 and PNM1, which both have nucleus localized signals [5,40,41]. To determine the subcellular localization of PPR18, the 550 amino acid residues of the N-terminal PPR18 were fused to the green fluorescent protein (GFP) in the binary vector pGWB5, then transiently expressed in the tobacco leaves via *Agrobacterium* EHA105 infiltration. Confocal laser-scanning microscopy revealed that the strong green fluorescence signals of PPR18^N550^-GFP are merged with the red signals of MitoTracker (Figure 1C), indicating that PPR18 is localized in mitochondria.

### 2.2. Embryo and Endosperm Development Are Arrested in ppr18

**Figure 2:**
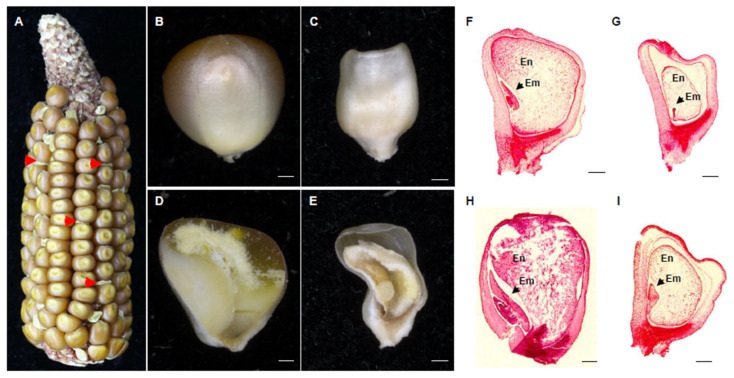
Mutant *ppr18-1* kernels abort early in embryogenesis and endosperm development. (**A**) The mature ear segregates 3:1 for wild-type (WT) and *ppr18-1* kernels at 35 days after pollination (DAP). The red arrows indicate the *ppr18-1* kernels. (**B**,**C**) Mature wild-type (**B**) and *ppr18-1* kernels (**C**) from (**A**). (**D**,**E**) Dissection of mature wild-type (**D**) and *ppr18-1* kernels (**E**). (**F**–**I**) Paraffin sections of wild-type (**F**,**H**) and *ppr18-1* kernels (**G**,**I**) at 9 DAP and 14 DAP. Wild-type kernels at 9 DAP (**F**) and 14 DAP (**H**); *ppr18-1* kernels at 9 DAP (**G**) and 14 DAP (**I**). En, endosperm; Em, embryo. Scale bar, 1 mm in (**B**–**I**).

To characterize the function of PPR18, we isolated two independent *Mutator* (*Mu*) insertional mutants containing *Mu* insertions at 880 bp and 939 bp downstream from the start codon of *PPR18* from the UniformMu population in the inbred W22 genetic background, named *ppr18-1* and *ppr18-2*, respectively [42]. The selfed progeny of both *ppr18-1*/+ and *ppr18-2*/+ heterozygotes segregated at a 3:1 ratio of wild-type (WT) and *empty pericarp* (*emp*) kernels, suggesting that both mutants are monogenic nuclear recessive mutations and homozygous lethal (Figure 2A and Figure S2A). Co-segregation analysis of a small isolated population with 72 individuals was performed to test the linkage of *ppr18-1* by genomic PCR using *PPR18*-R1 and *Mu* TIR8 primers [43]. The results showed that all the self-pollinated progenies of *ppr18-1*/+ plants produced *emp* kernels, indicating that the *Mu* insertion is tightly linked to the mutation (Figure S3). Crosses between *ppr18-1*/+ and *ppr18-2*/+ heterozygotes produced heteroallelic progeny *ppr18-1*/*ppr18-2* with approximately 25% *emp* kernels (Figure S2B), confirming that the *ppr18* phenotype results from the disruption of GRMZM2G438456.

The developing kernels phenotype of WT and *ppr18-1* in the same segregating ear are compared in Figure 2B,C. The *ppr18-1* mutant kernels are remarkably smaller than the wild type, which exhibited pale, half-translucent, and collapsed appearance at 35 days after pollination (DAP; Figure 2B–E). Compared with the WT siblings in the same segregating ear, the embryo and endosperm development are arrested in the *ppr18-1* kernels (Figure 2B–E). To examine the developmental arrest of embryogenesis in *ppr18-1*, we examined the embryo and endosperm development process between *ppr18-1* and WT siblings in a segregating ear by light microscopy. At 9 DAP, the WT embryos reached the coleoptilar stage, whereas the *ppr18-1* embryos remained at the pre-embryo stage (Figure 2F,G). At 14 DAP, the WT embryos reached late embryogenesis stage, while the *ppr18-1* embryos stayed at the transition stage without any discernable differentiation (Figure 2H,I). These results indicate that the loss of *PPR18* severely arrests both embryo and endosperm development.

To assess the impact of the *Mu* insertion on the *PPR18* expression, we analyzed the transcript level of *PPR18* in two *ppr18* alleles by reverse transcription PCR (RT-PCR). Results showed that no transcript of *PPR18* was detected in both alleles (Figure S4A), indicating that both alleles are probably null mutations. In wild type, *PPR18* transcripts can be detected in all vegetative and reproductive tissues by quantitative real-time PCR (qRT-PCR; Figure S4B). Relative high mRNA expression of *PPR18* was in bract and low expression in root, flower and kernel at developmental stages, indicating that *PPR18* is a constitutively expressed gene throughout growth and development in maize, rather than a seed specific gene. As the mutants are embryo lethal, impacts on other tissues and during development cannot be determined.

### 2.3. Loss of PPR18 Affects Mitochondrial Respiratory Activity

**Figure 3:**
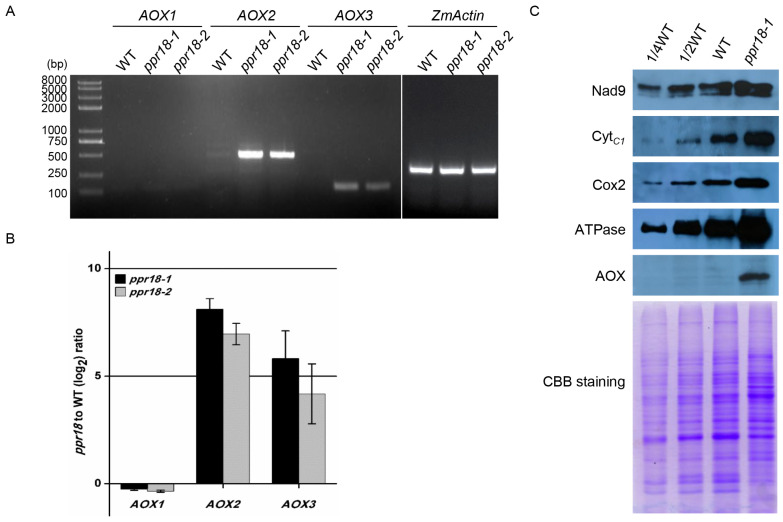
Expression of alternative oxidase (*AOX*) genes and analysis of protein expression abundance in the *PPR18* mutants. (**A**) The transcript levels of *AOX* genes in *ppr18-1* and *ppr18-2* kernels at 12 days after pollination (DAP). The expression levels were normalized to *ZmActin* (GRMZM2G126010). (**B**) qRT-PCR analysis of *AOX* gene expression in *ppr18-1* and *ppr18-2* kernels at 12 DAP. (**C**) Western blot analysis with antibodies against Nad9, Cyt*_C_*_1_, Cox2, ATPase a subunit, and AOX. Crude mitochondrial extracts from endosperm and embryo of *ppr18-1* and WT immature kernels at 11 DAP. CBB (Coomassie Brilliant Blue) staining demonstrates that equal amounts of mitochondrial proteins were loaded.

Previous reports showed the mutation of mitochondrion-localized PPR proteins with arrested seed development often suffers defects in mitochondrial respiration [44,45,46]. Thus, we investigated whether the loss of *PPR18* affects mitochondrial respiratory activity in maize by determining the respiratory activity, as shown by three mitochondrial respiratory rates, including total respiratory (V_t_), cytochrome respiratory capacity (V_cyt_), and alternative respiratory capacity (V_alt_). The ratio of V_cyt_/V_t_ was significantly reduced in the *ppr18-1* mutant compared with WT (Table 1), indicating that loss of *PPR18* resulted in a severe reduction of the cytochrome pathway and impaired mitochondrial respiration. Meanwhile, the ratio of V_alt_/V_t_ was markedly increased in *ppr18-1* (Table 1), supporting that alternative respiratory pathway was enhanced in the *ppr18-1* mutant. Moreover, we detected the expression of alternative oxidase (AOX) protein by Western blot assay using the specific antibody of AOX. Results showed that the abundance of AOX was increased drastically in the *ppr18-1* mutant compared to WT (Figure 3C), confirming that mutation of *PPR18* enhances the expression of alternative oxidases. The maize genome contains three *AOX* genes, *AOX1*, *AOX2*, and *AOX3*. Both RT-PCR and qRT-PCR assays showed that the expression of *AOX2* and *AOX3* was dramatically increased in the two *ppr18* alleles (Figure 3A,B), indicating that excessive accumulation of AOX is caused by upregulation of *AOX2* and *AOX3* expression in the *ppr18* alleles.

### 2.4. Loss of PPR18 Affects the Assembly and Activity of Complex I

**Figure 4:**
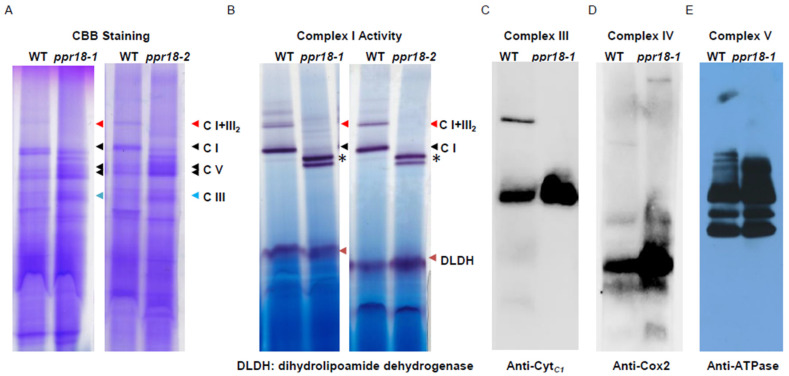
BN-PAGE analysis of mitochondrial complexes in *ppr18-1* and *ppr18-2* mutants. (**A**) About 130 mg of mitochondrial protein was loaded to a 3%–12% BN-PAGE. Blue native (BN) gels were stained with Coomassie brilliant blue (CBB). The positions of complex I, III, V, and super complex I + III_2_ are indicated. (**B**) In-gel NADH dehydrogenase activity of complex I. The activity of dihydrolipoamide dehydrogenase (DLDH) was used as a loading control. Asterisks indicate partially assembled complex I. (**C**–**E**) Accumulation of respiratory chain complex III (**C**), IV (**D**), and V (**E**) in *ppr18* mutant kernels. The BN gels were probed with antibodies against Cyt*_C_*_1_, Cox2, and ATPase (α-subunit).

The limited cytochrome pathway is closely relevant to the defective transfer electrons from mitochondrial respiratory complexes, complex I to IV [47,48]. To determine the assembly and abundance of mitochondrial respiratory complexes, we performed blue native (BN)-PAGE using crude mitochondria from *ppr18* alleles and WT maize kernels (Figure 4). As indicated by Coomassie Brilliant Blue (CBB) staining, complex III and V was substantially accumulated, whereas complex I and supercomplex I + III_2_ were depleted in both *ppr18* alleles compared to WT (Figure 4A), indicating that loss of *PPR18* affects the assembly of mitochondrial complex I. Furthermore, we analyzed the NADH dehydrogenase activity of complex I by in-gel NADH activity assay, which showed a consistent result with the CBB staining. As shown in Figure 4B, the dehydrogenase activity of complex I and supercomplex I + III_2_ were completely deficient in both *ppr18* alleles. Besides, we detected the assembly of complex III, IV, and V by Western blot analysis using the anti-Cyt*_C_*_1_, anti-Cox2, and anti-ATP synthase α-subunit antibody, respectively. Results showed that complex III, IV, and V were increased in the *ppr18-1* mutant (Figure 4C–E). Collectively, these results imply that PPR18 is important for the assembly and activity of mitochondrial complex I in maize.

In addition, we determined the abundance of the mitochondrial complex proteins in *ppr18-1* and WT maize kernels by Western blot analysis using antibodies against Nad9 (complex I), Cyt*_C_*_1_ (complex III), Cox2 (complex IV), and ATPase (complex V). As shown in Figure 3C, the protein abundance of Nad9, Cyt*_C_*_1_, Cox2, and ATPase was increased in *ppr18-1*, speculating that the lack of *PPR18* may enhance the expression of subunit from complex I, III, IV, and V in a feedback mechanism.

### 2.5. PPR18 Is Required for the Splicing of nad4 Intron 1

**Figure 5:**
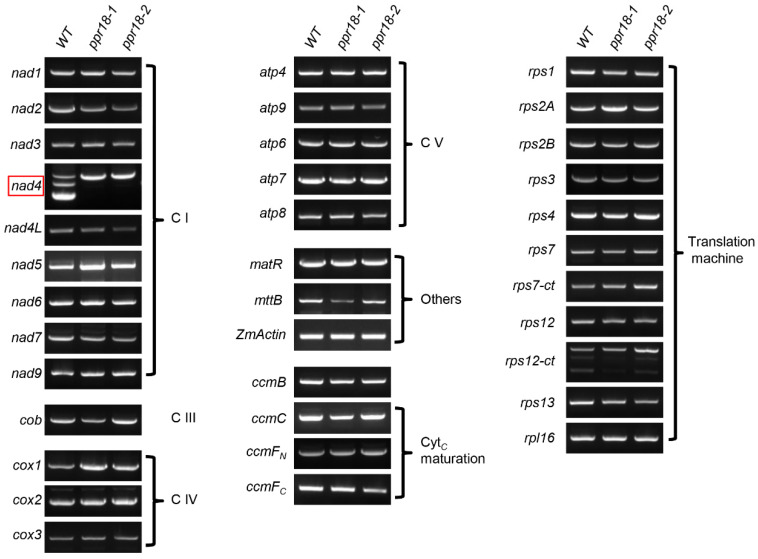
The *ppr18* mutants only affect the expression of mitochondrial *nad4* mature transcript. The transcript levels of 35 mitochondrion-encoded genes in *ppr18-1 and ppr18-2* mutant kernels. The RNA was isolated from the same ear segregating for WT and *ppr18* mutants at 13 days after pollination (DAP). The expression levels were normalized to *ZmActin* (GRMZM2G126010). The absence of *nad4* transcripts occurs in both *ppr18* alleles (red box indicated). C I: Complex I, C II: Complex II, C III: Complex III, C IV: Complex IV, C V: Complex V.

Previous study showed that most P-type PPR proteins function on intron splicing, RNA maturation, RNA stabilization or RNA cleavage in organelles [5]. To reveal the molecular function of PPR18, we analyzed the transcript levels of 35 mitochondrion-encoded genes between WT and *ppr18* alleles by RT-PCR and qRT-PCR. The results showed that the expression level of most mitochondrion-encoded genes was indistinguishable between the WT and *ppr18* alleles, only the expression of *nad4* was obviously different between WT and *ppr18* alleles (Figure 5 and Figure S5). The mature *nad4* transcript was not detectable in both *ppr18* alleles. Instead, a band larger than the mature *nad4* transcript was dramatically increased in both *ppr18* mutants (Figure 5). The sequencing results of the larger fragments showed that these fragments contain unspliced *nad4* intron 1. These results indicate that the loss-of-function in PPR18 abolishes the splicing of *nad4* intron 1 in mitochondria.

**Figure 6:**
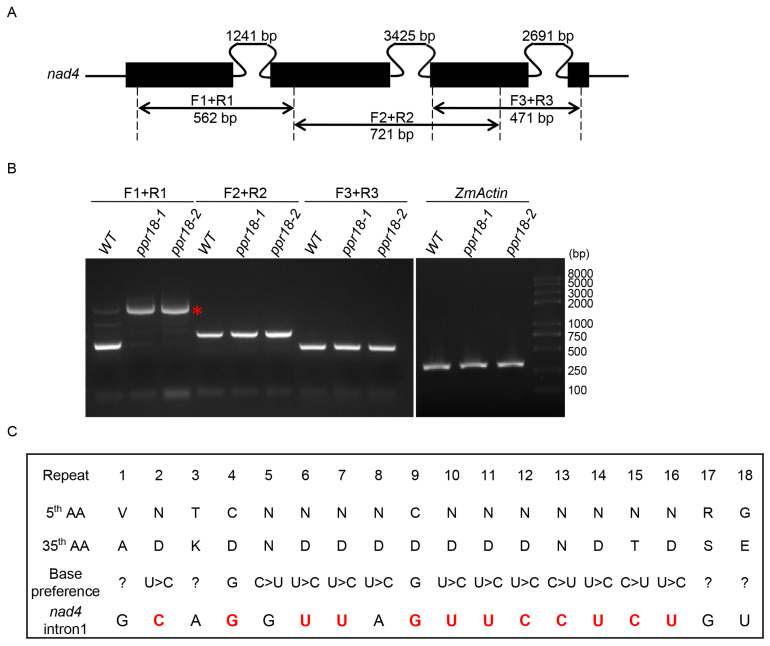
The *ppr18* mutants are impaired in the splicing of *nad4* intron 1. (**A**) Gene structure diagram of the maize *nad4* gene. Exons are shown as filled black boxes. All the three introns of *nad4* are *cis*-introns. The primers and expected amplification products using are indicated. F: Forward primer, R: Reverse primer. (**B**) RT-PCR analysis of the intron splicing of *nad4* introns using primers as indicated in (**A**). Asterisk indicates the unspliced PCR products of *nad4* intron 1. (**C**) Binding predictions for the PPR18 proteins on the respective targets *nad4* intron 1 referred to Barkan et al., 2012; Yin et al., 2013; and Gully et al., 2015. The amino acid (AA) residues at position 5th and 35th in PPR motifs 1–18 are indicated. Nucleotides matching the amino acid combination are indicated in red. “?” indicates an unidentified nucleotide.

**Figure 7:**
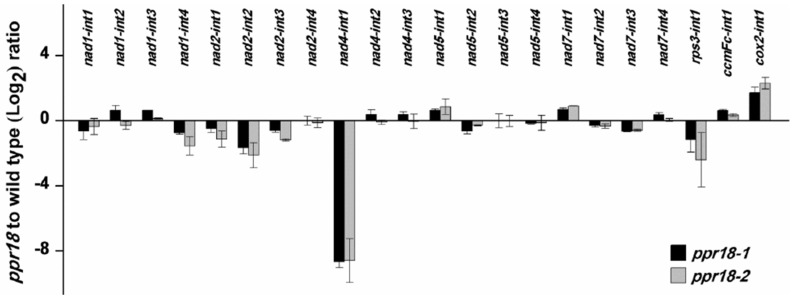
Splicing efficiency of mitochondrial introns in *ppr18* mutants. qRT-PCR analysis of the splicing efficiency of all 22 group II introns of maize mitochondrial genes in *ppr18* mutants. The ratio of spliced to unspliced fragments was used to measure splicing efficiency. Data are means (± SE) of three biological replicates.

The *nad4* precursor RNA contains three *cis*-splicing introns (Figure 6A). To confirm the function of *PPR18* on the *nad4* intron 1 splicing, we amplified fragments containing each of the three introns in the *nad4* transcript by RT-PCR using specific primers. As shown in Figure 6B, only the splicing of *nad4* intron 1 was impaired in both *ppr18* alleles. In addition, we analyzed the splicing efficiency of 22 group II introns in mitochondria by qRT-PCR. Results showed that the splicing efficiency of *nad4* intron 1 was dramatically decreased in the *ppr18* alleles (Figure 7). These data suggest that PPR18 is indeed required for the splicing of mitochondrial *nad4* intron 1. Previous report showed that PPR proteins bind specific RNA via a modular recognition code in which the nucleotide specificity primarily relies on combination at the 5th and 35th amino acid residues of each PPR motif [29,30,31]. Based on PPR recognition code, potential binding sites of PPR18 in mitochondrial *nad4* intron 1 were predicted (Figure 6C). Results showed that the nucleotides of *nad4* intron 1 are well aligned to the combinatorial codes. We predicted the secondary structure of *nad4* intron 1 and mapped the putative binding site of PPR18 in domain I of *nad4* intron 1 (Figure S6). A phylogenetic analysis based on genomic DNAs in the GenBank, the putative binding site of PPR18 in *nad4* intron 1 appeared to be highly conserved in both monocots and dicots (Figure S7).

### 2.6. PPR18 Does Not Show a Direct Interaction with DEK35, EMP8, and EMP602 in Yeast Two-Hybrid Assays

Previous studies reported that three PPR proteins DEK35, EMP8, and EMP602 are involved in the splicing of mitochondrial *nad4* intron 1 in maize [46,49,50]. In this study, PPR18 is also required for the splicing of *nad4* intron 1. To determine whether PPR18 interacts with DEK35, EMP8, and EMP602, we performed a yeast two-hybrid assay. Results showed that PPR18 has no directly physical interaction with these proteins (Figure S8).

## 3. Discussion

### 3.1. A Role of PPR18 on nad4 Intron 1 Splicing and the Assembly of Complex I

The maize mitochondria contain a total of 22 group II introns in 8 genes (*nad1*, *nad2*, *nad4*, *nad5*, *nad7*, *ccmFc*, *cox2*, and *rps3*) [4]. Some PPR proteins have been reported to be responsible for the splicing of group II introns in maize mitochondria (Table 2). Loss-of-function of these PPR proteins usually results in *empty pericarp* (*emp*) or *defective kernel* (*dek*) phenotype in maize. For example, disruption of *Dek2* and *Dek37* causes small kernels and delayed development, leading to a *dek* phenotype of maize [35,38]. The mutation of *Emp16* and *Emp10* severely arrests seed development, resulting in embryo lethality and an *emp* phenotype of maize [25,39]. Similarly, the loss of *PPR18* function severely arrests the embryo and endosperm development (Figure 2), leading to the *emp* phenotype, suggesting that PPR18 is crucial for maize kernel development.

As shown in Table 2, these PPR proteins (EMP8, EMP10, EMP11, EMP12, EMP16, EMP602, DEK2, DEK35, DEK37, DEK41, DEK43, PPR-SMR1, and PPR20) are reported to participate in the splicing of *nad1*, *nad2*, *nad4*, *nad5*, *nad7*, and *rps3* introns. These *nad* genes encode the core subunits of mitochondrial complex I and lack of these Nad proteins causes the disassembly and reduced activity of complex I in maize [51]. For example, EMP11 is responsible for the intron splicing of *nad1* and the dysfunction of *Emp11* affects the assembly and stability of mitochondrial complex I [34]. EMP12 and PPR20 are essential for the splicing of *nad2* introns. The mutation of EMP12 and PPR20 results in disassembly of mitochondrial complex I and a significant reduction in the dehydrogenase activity [36,37]. In this study, loss of *PPR18* impaired the *cis*-splicing of *nad4* intron 1 and the accumulation of mature *nad4* transcript (Figure 5), leading to severely defective assembly and activity of mitochondrial complex I in the *ppr18* mutants, suggesting the importance of PPR18 in the intron splicing of *nad4* transcript and the assembly and activity of mitochondrial complex I. The splicing of *nad4* intron 1 was dramatically reduced in *dek35*, and completely abolished in *emp8*, *emp602*, and *ppr18* [46,49,50], causing a deficiency in the mature *nad4* transcript and severely arrested embryo and endosperm development in maize. Together, these results indicate that expression of *nad4* is essential to the mitochondrial function and kernel development in maize. A co-evolution between the mitochondrial *nad4* intron 1 and the nuclear *PPR18* genes are implicated as indicated by the highly conserved sequences between PPR18 and its putative binding site in *nad4* intron 1 in both monocots and dicots (Figures S1 and S7).

In Figure 4, the in-gel NADH activity assay showed that two bands with a smaller size than mature complex I were produced in *ppr18* alleles, which are probably partially assembled complex I. Additionally, the two smaller size complexes had the dehydrogenase activity (Figure 4B), indicating the two partially assembled complex I are stable intermediate of the mitochondrial complex I assembly pathway in maize. Similar cases were also found in the *dek35*, *emp602*, *dek41*, and *dek43* mutants, which are defective in the splicing of *nad4* introns and [49,50,52,53], supporting that Nad4 is assembled into the complex I at a late stage. As reported in *Arabidopsis*, Nad4 is located in the P_D_ module of the membrane arm of mitochondrial complex I, which is associated with assembled intermediate to form the mature complex I [51], indicating that Nad4 plays a crucial role during the last phase of the complex I assembly process both in monocots and dicots.

Previous reports showed that impairments in the electron transfer chain (ETC) can enhance AOX pathway in mitochondria [54,55,56]. In the *ppr18* alleles, the ratio of V_alt_/V_t_, the abundance of AOX protein, and the expression of *AOX2* and *AOX3* transcripts were notably increased (Figure 3), indicating that AOX pathway is significantly enhanced in *ppr18*. A retrograde signalling pathway is strongly implicated as the AOXs are nucleus-encoded proteins.

### 3.2. Multiple Splicing Factors Participate in the Splicing of nad4 Intron 1

The defective splicing of *nad4* intron 1 was firstly reported in *Nicotiana sylvestris nms1* mutant [57]. In maize, defects of splicing of *nad4* intron 1 have been found in some *ppr* mutants, e.g., *dek35*, *emp8*, and *emp602* [46,49,50]. Our data show that PPR18 also specifically functions on the splicing of *nad4* intron 1 in maize. These splicing factors share common intron target with PPR18, implying that splicing of a single intron requires multiple splicing factors. Based on the yeast two-hybrid analyses, however, PPR18 does not directly interact with DEK35, EMP8, and EMP602 (Figure S8).

It is possible that these PPR proteins function independently by binding to the specific sequences of *nad4* intron 1 to maintain a splicing-competent structure, and these proteins do not have protein interactions with each other. Proteins could promote group II intron folding to form the native structure [58]. PPR proteins are RNA binding proteins that could guide intron folding by sequence-specific interactions [59]. PPR18 may play a role in folding of *nad4* intron 1 to participate in the intron splicing of *nad4* transcript.

As the intron splicing mechanism in mitochondria is not clear yet, all co-factors may have not been identified. The possibility of a ribonucleoprotein complex similar to the nuclear spliceosome exists for some introns. Thus, it is possible that PPR18 may interact with other splicing co-factors. The tested PPR proteins may interact with some key splicing factors exist in splicing complexes to mediate splicing of *nad4* intron 1. For example, PPR-SMR1 interacts with Zm-mCSF1 to participate in the splicing of several mitochondrial group II introns, speculating that PPR-SMR1 and Zm-mCSF1 might be the core splicing factors to mediate multiply group II introns splicing [60]. Further studies are necessary to unravel the splicing mechanism of plant organellar group II introns.

## 4. Materials and Methods

### 4.1. Plant Materials

The *ppr18* alleles (UFMu-06715 and UFMu-11033) were obtained from the Maize Genetics Cooperation Stock Center (Urbana, IL, USA). For developmental analyses and population generation, the maize plants were cultivated in the experimental filed of Shandong University in Qingdao. Tobacco (*Nicotiana tabacum*) was grown in climate chambers at 25 °C/22 °C day/night on a 12 h light/12 h dark regime.

### 4.2. Subcellular Localization

To express PPR18 ^N550^-GFP fusion proteins, the amplified PPR18 N-terminal coding sequence (1650 bp) was cloned into binary vector pGWB5 driven by the 35S promoter. *Agrobacterium tumefaciens* strain (EHA105) harboring this construct was infiltrated into tobacco (*Nicotiana tabacum*) leaf epidermis with a syringe, as previously described [61]. After incubation at 24 °C for 24–30 h, the GFP signals were observed and imaged using ZEISS LSM 880 confocal microscope (Carl-Zeiss, Jena, Germany). MitoTracker Red (ThermoFisher Scientific, Waltham, MA, USA) was used as the mitochondrion marker with a working concentration of 100 nM.

### 4.3. Light Microscopy of Cytological Sections

*ppr18-1*/+ heterozygotes were identified by PCR with *Mu* primer TIR8 and gene primer *PPR18*-R1. Immature WT and *ppr18-1* kernels were harvested from the self-pollinated heterozygous segregating ear at 9 and 14 days after pollination (DAP). The fixed material and sections were performed as described previously [62]. Paraffin sections were stained with 1% Johansen’s Safranin O and imaged with a stereo microscope (Carl-Zeiss, Jena, Germany).

### 4.4. RNA Extraction, RT-PCR, and qRT-PCR

Total RNA was extracted from fresh kernels by removing the pericarp using TRIzol reagent (ThermoFisher Scientific, Waltham, MA, USA). After DNaseI digestion (NEB) to eliminate DNA contamination, reverse transcription was conducted with random primers according to the manufacturer’s instructions (TransGen Company, Beijing, China). Quantitative real time PCR (qRT-PCR) was performed with using LightCycler 96 (Roche, Basel, Switzerland) with three biological replicates. The maize actin gene *ZmActin* (GRMZM2G126010) was used as the reference gene. For functional analysis of *PPR18*, RT-PCR and qRT-PCR were performed with primers as previously described [45,49].

### 4.5. Measurements of Respiration Rate

The respiration rates were determined according to the previous report with some modifications [63]. The respiratory activities were measured in a reaction medium (50 mM phosphate buffer, pH 6.8) at 25 °C in the dark using a Chlorolab II liquid oxygen electrode (Hansatech, King’s Lynn, UK, http://hansatech-instruments.com). The alternative pathway capacity (V_alt_) and cytochrome pathway capacity (V_cyt_) are defined as O_2_ uptake rate in the presence of 2 mM potassium cyanide (KCN; Sigma-Aldrich, St. Louis, MO, USA, catalog number: 207810) and 2 mM salicylhydroxamic acid (SHAM; Sigma-Aldrich, St. Louis, MO, USA, catalog number: S607), respectively. All the respiration rates were indicated by the oxygen consumption of nmol O_2_ min^−1^ g^−1^ fresh weight of the maize kernels.

### 4.6. Blue Native (BN)-PAGE and Complex I Activity Assay

Crude mitochondrial proteins were extracted from immature maize kernels with the pericarp removed at 11 DAP. Blue native (BN)-PAGE and measurement of NADH dehydrogenase activity were performed as previously described [64]. Of maize mitochondrial proteins 130 µg was separated by 3%–12.5% gradient gel (ThermoFisher Scientific, Waltham, MA, USA). The gel strips were stained by Coomassie Blue R-250 and assayed for complex I activity in nitroblue tetrazolium (NBT)-NADH buffer (25 mg of NBT, 100 µL of NADH (10 mg mL^−1^), and 10 mL of 5 mM Tris–HCl, pH 7.4). The gel strips were transferred to the nitrocellulose membrane and Western blotting with specific antibodies cytochrome *c*_1_ (Cyt*_C_*_1_, a gift from G. Schatz, University of Basel, Switzerland), *Arabidopsis* Cox2 (Agrisera, Vännäs, Sweden, http://www.agrisera.com), and ATPase (ATPase α-subunit, MBL Beijing Biotech, China) for detection of complex III, VI, and V, respectively [64].

### 4.7. Immunoblot Analysis

Proteins extracted from immature maize kernels were determined by 12.5% SDS-PAGE and transferred to the Polyvinylidene Fluoride (PVDF, GE healthcare, Freiburg im Breisgau, Germany) membrane and Western blotting using antiserum against Cyt*_C_*_1_ (1:5000), Cox2 (1:5000), ATPase (1:10,000), AOX (1:10,000) [25], and wheat Nad9 (1:3000) [65], as previously described [44]. The membrane was treated with ECL reagents (Pierce, ThermoFisher Scientific, Waltham, MA, USA). Signals were visualized on X-ray films (Kodak, Tokyo, Japan) and imaged using a Tanon-5200 system (Tanon, Shanghai, China).

### 4.8. Yeast Two-Hybrid Analysis

Yeast two-hybrid analysis was performed according to the manual of Matchmaker™ Gold Yeast Two-Hybrid System (Clontech, Mountain View, CA, USA). The coding sequence (CDS) without signal peptide sequences of PPR18, DEK35, EMP8, and EMP602 were cloned pGADT7 vector and pGBKT7 vector, respectively. The primer sequences are listed in Table S1. Combinations of plasmids were co-transformed into the yeast strain Y2H Gold (Clontech, Mountain View, CA, USA) and placed on SD/–Leu/–Trp (Minimal Media Double Dropouts, DDO) mediums and growth of diploid yeast colonies on SD/–Ade/–His/–Leu/–Trp (Minimal Media Quadruple Dropouts, QDO) mediums for 4 days at 30 °C to reveal protein–protein interactions.

### 4.9. Prediction of PPR18 Binding Site

PPR motif prediction alignment analysis of PPR18 protein was used by algorithm TPRpred (http://tprpred.tebingen.mpg.de/tprpred). The alignment of PPR18 to its *nad4* intron 1 binding site was generated as follows the PPR codes [29,30,31]. The recognition nucleotides for the PPR18 protein were predicted by the arrangements of the amino acids at position 5th and 35th of each PPR repeat from PPR18. The respective recognition nucleotides were listed and aligned with *nad4* intron 1 to find the best matched nucleotide sites as potential binding sites of PPR18 in mitochondrial *nad4* intron 1.

### 4.10. Phylogenetic Analysis

The full-length amino acid sequences of PPR18, the putative binding site of PPR18 in *nad4* intron 1, and their orthologs in plant species were downloaded from NCBI database (https://blast.ncbi.nlm.nih.gov). The phylogenetic tree was constructed using MEGA7 software by the maximum likelihood method [66].

## 5. Conclusions

In this study, we characterized a maize seed mutant *ppr18*, which exhibits an arrested embryo and endosperm development phenotype. Through a molecular characterization of the *PPR18* gene, we elucidated its function in the *cis*-splicing of *nad4* intron 1 in mitochondria and seed development in maize. The lack of splicing of *nad4* intron 1 results in the absence of *nad4* transcript, leading to severely reduced assembly and activity of mitochondrial complex I. The profiles of complex I assembly, activity, and component accumulation in the *ppr18* mutants shed lights to the assembly process of complex I in maize. Despite PPR proteins have been reported in intron splicing, our study provides additional information on a new PPR protein in intron splicing, complex I assembly, and its essential role in maize seed development.

## Figures and Tables

**Table 1:** Alteration of the respiration rate of the wild type (WT) and *ppr18-1* kernels.

	Respiration Rate (nmol O_2_ min^−1^ g^−1^ Fresh Weight)				
	V_t_	V_alt_	V_cyt_	V_alt_/V_t_ (%)	V_cyt_/V_t_ (%)
WT	824.96 ± 77.23	155.90 ± 14.10	734.30 ± 60.97	18.90	89.01
*ppr18-1*	175.62 ± 2.85	136.61 ± 6.55	39.00 ± 4.26	77.79	22.21

Mitochondrial total respiration rate (V_t_), the alternative pathway (V_alt_), and the capacity of the cytochrome pathway (V_cyt_) were indicated by the oxygen consumption of nmol O_2_ min^−1^ g^−1^ fresh weight of the maize kernels at 11 DAP using a Clark-type oxygen electrode. Data are mean values ± SEs from three independent biological samples.

**Table 2:** List of mitochondrion-localized PPR proteins are involved in intron splicing of mitochondrial genes in maize.

Protein	Target Transcript	Reference
EMP16	*nad2*-int4	(Xiu et al., 2016)
EMP10	*nad2*-int1	(Cai et al., 2017)
DEK35	*nad4*-int1	(Chen et al., 2017)
DEK2	*nad1*-int1	(Qi et al., 2017)
EMP11	*nad1*-int1, 2, 3, 4	(Ren et al., 2017)
DEK37	*nad2*-int1	(Dai et al., 2018)
EMP8	*nad1*-int4, *nad2*-int1, *nad4*-int1	(Sun et al., 2018)
EMP12	*nad2*-int1, 2, 4	(Sun et al., 2019)
DEK41/DEK43	*nad4*-int3	(Ren et al., 2019; Zhu et al., 2019)
PPR-SMR1	*nad1*-int1, 2, 3, 4, *nad2*-int1, 2, 3, 4, *nad4*-int1, 2, 3, *nad5*-int1, 3, 4, *nad7*-int2, *rps3*-int1	(Chen et al., 2019)
EMP602	*nad4*-int1, 3	(Ren et al., 2019)
PPR20	*nad2*-int3	(Yang et al., 2019)
PPR18	*nad4*-int1	This study

## Supplementary Materials

Supplementary materials can be found at https://www.mdpi.com/1422-0067/21/11/4047/s1, Figure S1: Phylogenetic analysis of PPR18 homologs, Figure S2: Phenotypes of the *ppr18-2* mutant and the ear of *ppr18-1* × *ppr18-2*, Figure S3: The linkage analysis of the *Mu* insertion in *PPR18-1* and the phenotype in the selfed progeny, Figure S4: Expression of *PPR18*, Figure S5: Transcript levels of the 35 mitochondrial genes in *ppr18* alleles in developing kernels, Figure S6: The predicted secondary structure of maize *nad4* intron 1 and the location of the putative binding site, Figure S7: Alignment of the putative binding site of PPR18 in *nad4* intron 1 in different plant species, Figure S8: Interaction assay of PPR18 and the related splicing factors, Table S1: Primers used in this study.

## References

[1] Timmis J.N., Ayliffe M.A., Huang C.Y., Martin W. (2004). Endosymbiotic gene transfer: Organelle genomes forge eukaryotic chromosomes. Nat. Rev. Genet..

[2] Unseld M., Marienfeld J.R., Brandt P., Brennicke A. (1997). The mitochondrial genome of *Arabidopsis thaliana* contains 57 genes in 366,924 nucleotides. Nat. Genet..

[3] Notsu Y., Masood S., Nishikawa T., Kubo N., Akiduki G., Nakazono M., Hirai A., Kadowaki K. (2002). The complete sequence of the rice (*Oryza sativa L.*) mitochondrial genome: Frequent DNA sequence acquisition and loss during the evolution of flowering plants. Mol. Genet. Genomics.

[4] Clifton S.W., Minx P., Fauron C.M., Gibson M., Allen J.O., Sun H., Thompson M., Barbazuk W.B., Kanuganti S., Tayloe C. (2004). Sequence and comparative analysis of the maize NB mitochondrial genome. Plant Physiol..

[5] Barkan A., Small I. (2014). Pentatricopeptide repeat proteins in plants. Annu. Rev. Plant Biol..

[6] Delannoy E., Stanley W.A., Bond C.S., Small I.D. (2007). Pentatricopeptide repeat (PPR) proteins as sequence-specificity factors in post-transcriptional processes in organelles. Biochem. Soc. Trans..

[7] Kramer M.C., Anderson S.J., Gregory B.D. (2018). The nucleotides they are a-changin’: Function of RNA binding proteins in post-transcriptional messenger RNA editing and modification in *Arabidopsis*. Curr. Opin. Plant Biol..

[8] Bonen L. (2008). *Cis*- and *trans*-splicing of group II introns in plant mitochondria. Mitochondrion.

[9] Lambowitz A.M., Zimmerly S. (2004). Mobile group II introns. Annu. Rev. Genet..

[10] Cech T.R. (1990). Self-splicing of group I introns. Annu. Rev. Biochem..

[11] Zoschke R., Nakamura M., Liere K., Sugiura M., Borner T., Schmitz-Linneweber C. (2010). An organellar maturase associates with multiple group II introns. Proc. Natl. Acad. Sci. USA.

[12] Vogel J., Börner T., Hess W.R. (1999). Comparative analysis of splicing of the complete set of chloroplast group II introns in three higher plant mutants. Nucleic Acids Res..

[13] Cohen S., Zmudjak M., Colas des Francs-Small C., Malik S., Shaya F., Keren I., Belausov E., Many Y., Brown G.G., Small I. (2014). nMAT4, a maturase factor required for *nad1* pre-mRNA processing and maturation, is essential for holocomplex I biogenesis in *Arabidopsis* mitochondria. Plant J..

[14] Keren I., Tal L., des Francs-Small C.C., Araujo W.L., Shevtsov S., Shaya F., Fernie A.R., Small I., Ostersetzer-Biran O. (2012). nMAT1, a nuclear-encoded maturase involved in the *trans*-splicing of *nad1* intron 1, is essential for mitochondrial complex I assembly and function. Plant J..

[15] Keren I., Bezawork-Geleta A., Kolton M., Maayan I., Belausov E., Levy M., Mett A., Gidoni D., Shaya F., Ostersetzer-Biran O. (2009). AtnMat2, a nuclear-encoded maturase required for splicing of group-II introns in *Arabidopsis* mitochondria. RNA.

[16] Till B., Schmitz-Linneweber C., Williams-Carrier R., Barkan A. (2001). CRS1 is a novel group II intron splicing factor that was derived from a domain of ancient origin. RNA.

[17] Zmudjak M., Colas des Francs-Small C., Keren I., Shaya F., Belausov E., Small I., Ostersetzer-Biran O. (2013). mCSF1, a nucleus-encoded CRM protein required for the processing of many mitochondrial introns, is involved in the biogenesis of respiratory complexes I and IV in *Arabidopsis*. New Phytol..

[18] Asakura Y., Galarneau E., Watkins K.P., Barkan A., van Wijk K.J. (2012). Chloroplast RH3 DEAD box RNA helicases in maize and *Arabidopsis* function in splicing of specific group II introns and affect chloroplast ribosome biogenesis. Plant Physiol..

[19] Bobik K., McCray T.N., Ernest B., Fernandez J.C., Howell K.A., Lane T., Staton M., Burch-Smith T.M. (2017). The chloroplast RNA helicase ISE2 is required for multiple chloroplast RNA processing steps in *Arabidopsis thaliana*. Plant J..

[20] Hammani K., Barkan A. (2014). An mTERF domain protein functions in group II intron splicing in maize chloroplasts. Nucleic Acids Res..

[21] Hsu Y.W., Wang H.J., Hsieh M.H., Hsieh H.L., Jauh G.Y. (2014). *Arabidopsis* mTERF15 is required for mitochondrial *nad2* intron 3 splicing and functional complex I activity. PLoS ONE.

[22] Colas des Francs-Small C., Kroeger T., Zmudjak M., Ostersetzer-Biran O., Rahimi N., Small I., Barkan A. (2012). A PORR domain protein required for *rpl2* and *ccmF_C_* intron splicing and for the biogenesis of *c*-type cytochromes in *Arabidopsis* mitochondria. Plant J..

[23] Kroeger T.S., Watkins K.P., Friso G., van Wijk K.J., Barkan A. (2009). A plant-specific RNA-binding domain revealed through analysis of chloroplast group II intron splicing. Proc. Natl. Acad. Sci. USA.

[24] Kuhn K., Carrie C., Giraud E., Wang Y., Meyer E.H., Narsai R., des Francs-Small C.C., Zhang B., Murcha M.W., Whelan J. (2011). The RCC1 family protein RUG3 is required for splicing of *nad2* and complex I biogenesis in mitochondria of *Arabidopsis thaliana*. Plant J..

[25] Xiu Z., Sun F., Shen Y., Zhang X., Jiang R., Bonnard G., Zhang J., Tan B.C. (2016). EMPTY PERICARP16 is required for mitochondrial *nad2* intron 4 *cis*-splicing, complex I assembly and seed development in maize. Plant J..

[26] Schmitz-Linneweber C., Williams-Carrier R.E., Williams-Voelker P.M., Kroeger T.S., Vichas A., Barkan A. (2006). A pentatricopeptide repeat protein facilitates the *trans*-splicing of the maize chloroplast *rps12* pre-mRNA. Plant Cell.

[27] Lurin C., Andres C., Aubourg S., Bellaoui M., Bitton F., Bruyere C., Caboche M., Debast C., Gualberto J., Hoffmann B. (2004). Genome-wide analysis of *Arabidopsis* pentatricopeptide repeat proteins reveals their essential role in organelle biogenesis. Plant Cell.

[28] Fujii S., Small I. (2011). The evolution of RNA editing and pentatricopeptide repeat genes. New Phytol..

[29] Barkan A., Rojas M., Fujii S., Yap A., Chong Y.S., Bond C.S., Small I. (2012). A combinatorial amino acid code for RNA recognition by pentatricopeptide repeat proteins. PLoS Genet..

[30] Gully B.S., Cowieson N., Stanley W.A., Shearston K., Small I.D., Barkan A., Bond C.S. (2015). The solution structure of the pentatricopeptide repeat protein PPR10 upon binding *atpH* RNA. Nucleic Acids Res..

[31] Yin P., Li Q., Yan C., Liu Y., Liu J., Yu F., Wang Z., Long J., He J., Wang H.W. (2013). Structural basis for the modular recognition of single-stranded RNA by PPR proteins. Nature.

[32] Burger G., Gray M.W., Lang B.F. (2003). Mitochondrial genomes: Anything goes. Trends Genet..

[33] Berrisford J.M., Sazanov L.A. (2009). Structural basis for the mechanism of respiratory complex I. J. Biol. Chem..

[34] Ren X., Pan Z., Zhao H., Zhao J., Cai M., Li J., Zhang Z., Qiu F. (2017). EMPTY PERICARP11 serves as a factor for splicing of mitochondrial *nad1* intron and is required to ensure proper seed development in maize. J. Exp. Bot..

[35] Qi W., Yang Y., Feng X., Zhang M., Song R. (2017). Mitochondrial function and maize kernel development requires Dek2, a pentatricopeptide repeat protein involved in *nad1* mRNA splicing. Genetics.

[36] Sun F., Xiu Z., Jiang R., Liu Y., Zhang X., Yang Y.Z., Li X., Zhang X., Wang Y., Tan B.C. (2019). The mitochondrial pentatricopeptide repeat protein EMP12 is involved in the splicing of three *nad2* introns and seed development in maize. J. Exp. Bot..

[37] Yang Y.Z., Ding S., Wang Y., Wang H.C., Liu X.Y., Sun F., Xu C., Liu B., Tan B.C. (2020). PPR20 is required for the *cis*-splicing of mitochondrial *nad2* intron 3 and seed development in maize. Plant Cell Physiol..

[38] Dai D., Luan S., Chen X., Wang Q., Feng Y., Zhu C., Qi W., Song R. (2018). Maize *Dek37* encodes a P-type PPR protein that affects *cis*-splicing of mitochondrial *nad2* intron 1 and seed development. Genetics.

[39] Cai M., Li S., Sun F., Sun Q., Zhao H., Ren X., Zhao Y., Tan B.C., Zhang Z., Qiu F. (2017). *Emp10* encodes a mitochondrial PPR protein that affects the *cis*-splicing of *nad2* intron 1 and seed development in maize. Plant J..

[40] Ding Y.H., Liu N.Y., Tang Z.S., Liu J., Yang W.C. (2006). *Arabidopsis* GLUTAMINE-RICH PROTEIN23 is essential for early embryogenesis and encodes a novel nuclear PPR motif protein that interacts with RNA polymerase II subunit III. Plant Cell.

[41] Hammani K., Gobert A., Hleibieh K., Choulier L., Small I., Giegé P. (2011). An *Arabidopsis* dual-Localized pentatricopeptide repeat protein interacts with nuclear proteins involved in gene expression regulation. Plant Cell.

[42] McCarty D.R., Settles A.M., Suzuki M., Tan B.C., Latshaw S., Porch T., Robin K., Baier J., Avigne W., Lai J. (2005). Steady-state transposon mutagenesis in inbred maize. Plant J..

[43] Tan B.C., Chen Z., Shen Y., Zhang Y., Lai J., Sun S.S. (2011). Identification of an active new mutator transposable element in maize. G3 (Bethesda).

[44] Sun F., Wang X., Bonnard G., Shen Y., Xiu Z., Li X., Gao D., Zhang Z., Tan B.C. (2015). *Empty pericarp7* encodes a mitochondrial E-subgroup pentatricopeptide repeat protein that is required for *ccmF_N_* editing, mitochondrial function and seed development in maize. Plant J..

[45] Li X.J., Zhang Y.F., Hou M., Sun F., Shen Y., Xiu Z.H., Wang X., Chen Z.L., Sun S.S., Small I. (2014). *Small kernel 1* encodes a pentatricopeptide repeat protein required for mitochondrial *nad7* transcript editing and seed development in maize (*Zea mays*) and rice (*Oryza sativa*). Plant J..

[46] Sun F., Zhang X., Shen Y., Wang H., Liu R., Wang X., Gao D., Yang Y.Z., Liu Y., Tan B.C. (2018). The pentatricopeptide repeat protein EMPTY PERICARP8 is required for the splicing of three mitochondrial introns and seed development in maize. Plant J..

[47] Acin-Perez R., Fernandez-Silva P., Peleato M.L., Perez-Martos A., Enriquez J.A. (2008). Respiratory active mitochondrial supercomplexes. Mol. Cell.

[48] Millar A.H., Whelan J., Soole K.L., Day D.A. (2011). Organization and regulation of mitochondrial respiration in plants. Annu. Rev. Plant Biol..

[49] Chen X., Feng F., Qi W., Xu L., Yao D., Wang Q., Song R. (2017). *Dek35* encodes a PPR protein that affects *cis*-splicing of mitochondrial *nad4* intron 1 and seed development in maize. Mol. Plant.

[50] Ren Z., Fan K., Fang T., Zhang J., Yang L., Wang J., Wang G., Liu Y. (2019). Maize *empty pericarp602* encodes a P-type PPR protein that is essential for seed development. Plant Cell Physiol..

[51] Ligas J., Pineau E., Bock R., Huynen M.A., Meyer E.H. (2019). The assembly pathway of complex I in *Arabidopsis thaliana*. Plant J..

[52] Zhu C., Jin G., Fang P., Zhang Y., Feng X., Tang Y., Qi W., Song R. (2019). Maize pentatricopeptide repeat protein DEK41 affects *cis*-splicing of mitochondrial *nad4* intron 3 and is required for normal seed development. J. Exp. Bot..

[53] Ren R.C., Wang L.L., Zhang L., Zhao Y.J., Wu J.W., Wei Y.M., Zhang X.S., Zhao X.Y. (2020). DEK43 is a P-type PPR protein responsible for the *cis*-splicing of *nad4* in maize mitochondria. J. Integr. Plant Biol..

[54] De Longevialle A.F., Meyer E.H., Andres C., Taylor N.L., Lurin C., Millar A.H., Small I.D. (2007). The pentatricopeptide repeat gene OTP43 is required for *trans*-splicing of the mitochondrial *nad1* intron 1 in *Arabidopsis thaliana*. Plant Cell.

[55] Karpova O.V., Kuzmin E.V., Elthon T.E., Newton K.J. (2002). Differential expression of alternative oxidase genes in maize mitochondrial mutants. Plant Cell.

[56] Toda T., Fujii S., Noguchi K., Kazama T., Toriyama K. (2012). Rice *MPR25* encodes a pentatricopeptide repeat protein and is essential for RNA editing of *nad5* transcripts in mitochondria. Plant J..

[57] Brangeon J., Sabar M., Gutierres S., Combettes B., Bove J., Gendy C., Chétrit P., Des Francs-Small C.C., Pla M., Vedel F. (2000). Defective splicing of the first *nad4* intron is associated with lack of several complex I subunits in the *Nicotiana sylvestris* NMS1 nuclear mutant. Plant J..

[58] Barkan A. (2004). Intron splicing in plant organelles. Mol. Biol. Biotechnol. Plant Organelles.

[59] Brown G.G., Colas des Francs-Small C., Ostersetzer-Biran O. (2014). Group II intron splicing factors in plant mitochondria. Front. Plant Sci..

[60] Chen Z., Wang H.C., Shen J., Sun F., Wang M., Xu C., Tan B.C. (2019). PPR-SMR1 is required for the splicing of multiple mitochondrial introns, interacts with Zm-mCSF1, and is essential for seed development in maize. J. Exp. Bot..

[61] Shen Y., Li C., McCarty D.R., Meeley R., Tan B.C. (2013). *Embryo defective12* encodes the plastid initiation factor 3 and is essential for embryogenesis in maize. Plant J..

[62] Zhang Y.F., Hou M.M., Tan B.C. (2013). The requirement of WHIRLY1 for embryogenesis is dependent on genetic background in maize. PLoS ONE.

[63] Wang X.M., Chang N., Bi Y.R., Tan B.C. (2015). Measurement of mitochondrial respiration rate in maize (*Zea mays*) leaves. Bio-Protocol.

[64] Meyer E.H., Tomaz T., Carroll A.J., Estavillo G., Delannoy E., Tanz S.K., Small I.D., Pogson B.J., Millar A.H. (2009). Remodeled respiration in *ndufs4* with low phosphorylation efficiency suppresses *Arabidopsis* germination and growth and alters control of metabolism at night. Plant Physiol..

[65] Lamattina L., Gonzalez D., Gualberto J., Grienenberger J.M. (1993). Higher plant mitochondria encode an homologue of the nuclear-encoded 30-kDa subunit of bovine mitochondrial complex I. Eur. J. Biochem..

[66] Kumar S., Stecher G., Tamura K. (2016). MEGA7: Molecular evolutionary genetics analysis version 7.0 for bigger datasets. Mol. Biol. Evol..

