# Supplementary material for: The Mitochondrial Pentatricopeptide Repeat Protein PPR18 Is Required for the cis-Splicing of nad4 Intron 1 and Essential to Seed Development in Maize
Source: Int J Mol Sci. 2020 Jun 5;21(11):4047. doi: 10.3390/ijms21114047 (PMC7312232; doi:10.3390/ijms21114047)
Supplement: Supplementary file 1 [file ijms-21-04047-s001.zip › PPR18 Supplemental Figs S1-S8.pptx]

## Slide 1
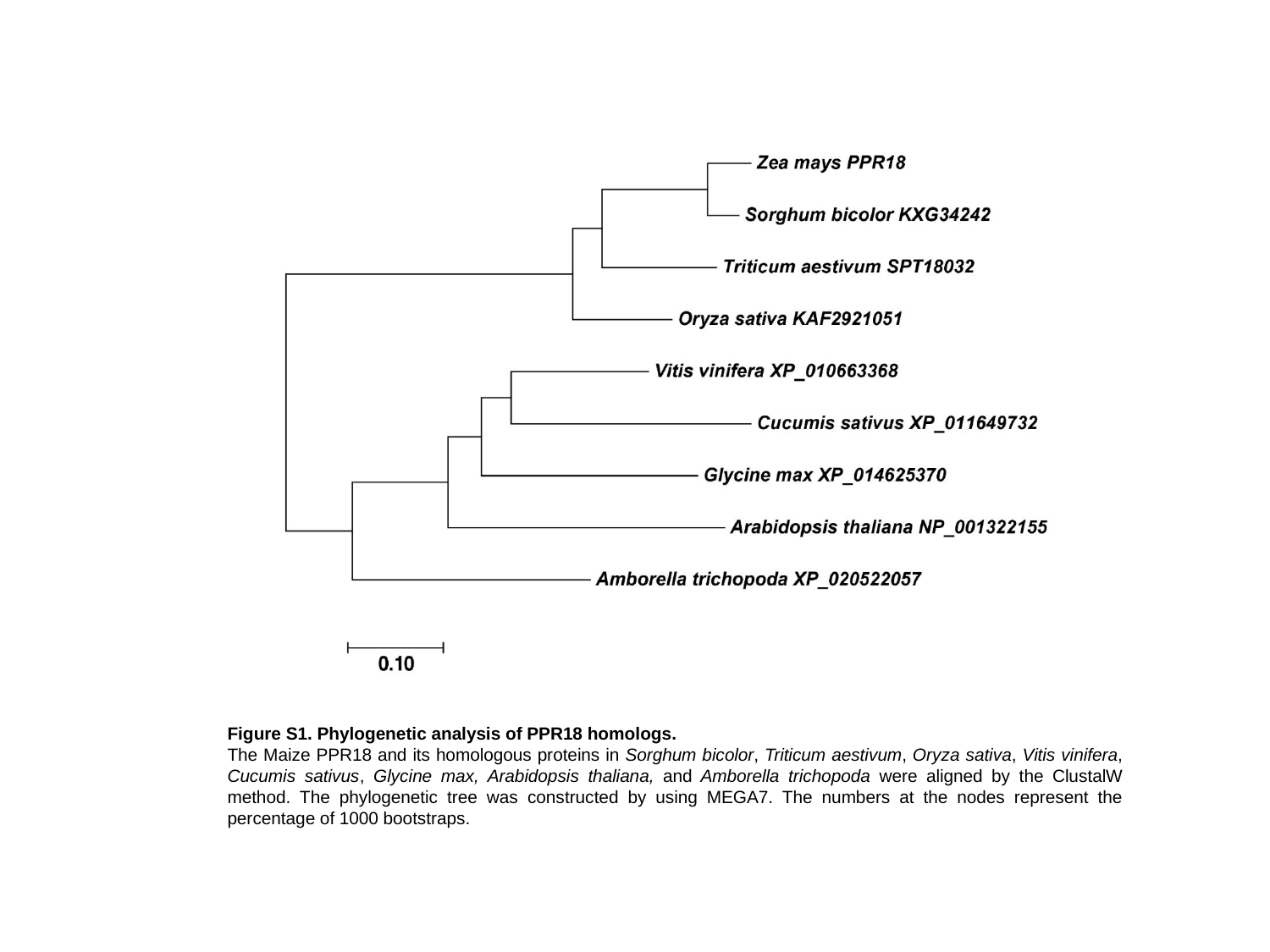

Figure S1. Phylogenetic analysis of PPR18 homologs.
The Maize PPR18 and its homologous proteins in Sorghum bicolor, Triticum aestivum, Oryza sativa, Vitis vinifera, Cucumis sativus, Glycine max, Arabidopsis thaliana, and Amborella trichopoda were aligned by the ClustalW method. The phylogenetic tree was constructed by using MEGA7. The numbers at the nodes represent the percentage of 1000 bootstraps.

## Slide 2
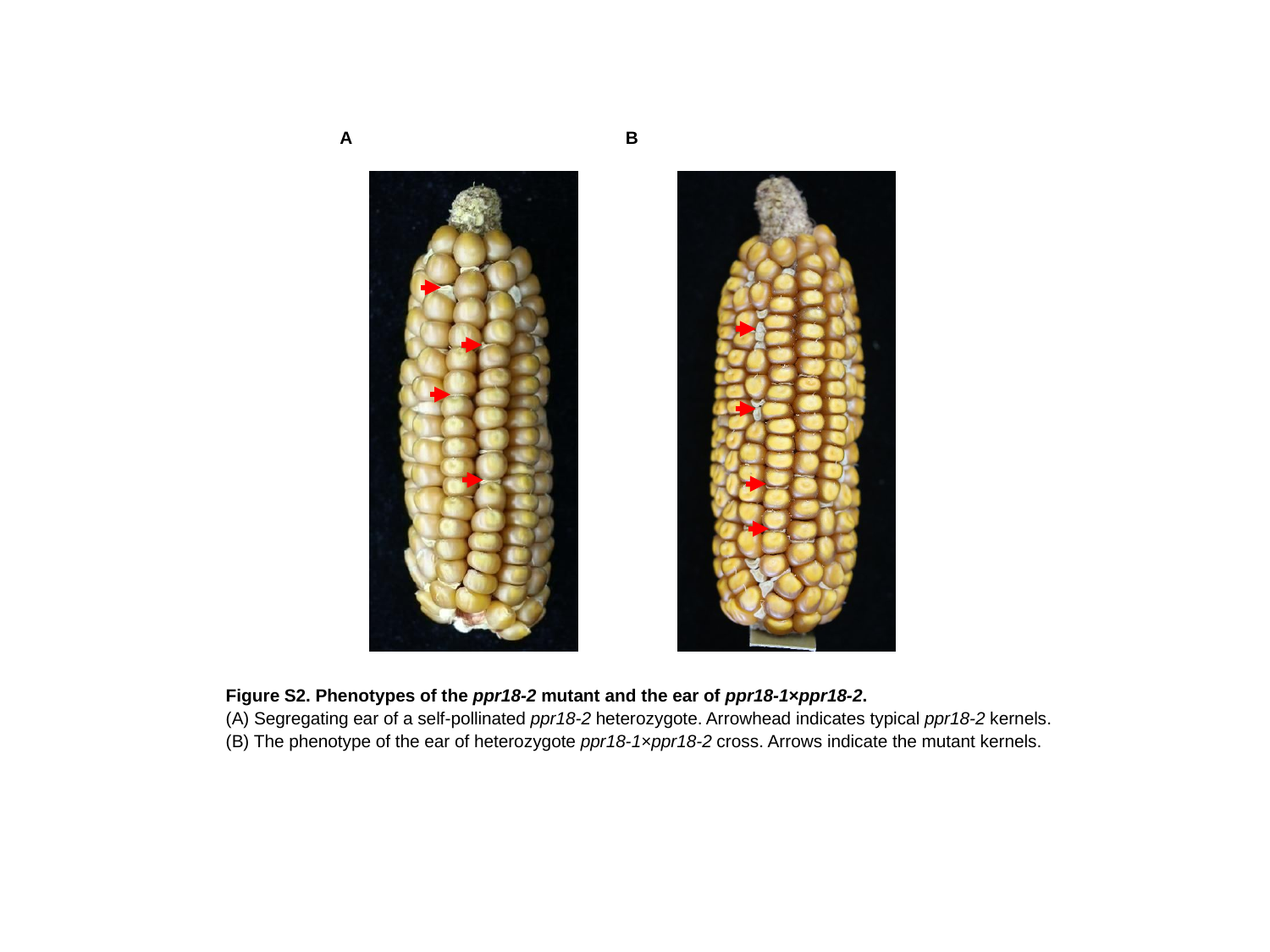

B
A
Figure S2. Phenotypes of the ppr18-2 mutant and the ear of ppr18-1×ppr18-2.
(A) Segregating ear of a self-pollinated ppr18-2 heterozygote. Arrowhead indicates typical ppr18-2 kernels.
(B) The phenotype of the ear of heterozygote ppr18-1×ppr18-2 cross. Arrows indicate the mutant kernels.

## Slide 3
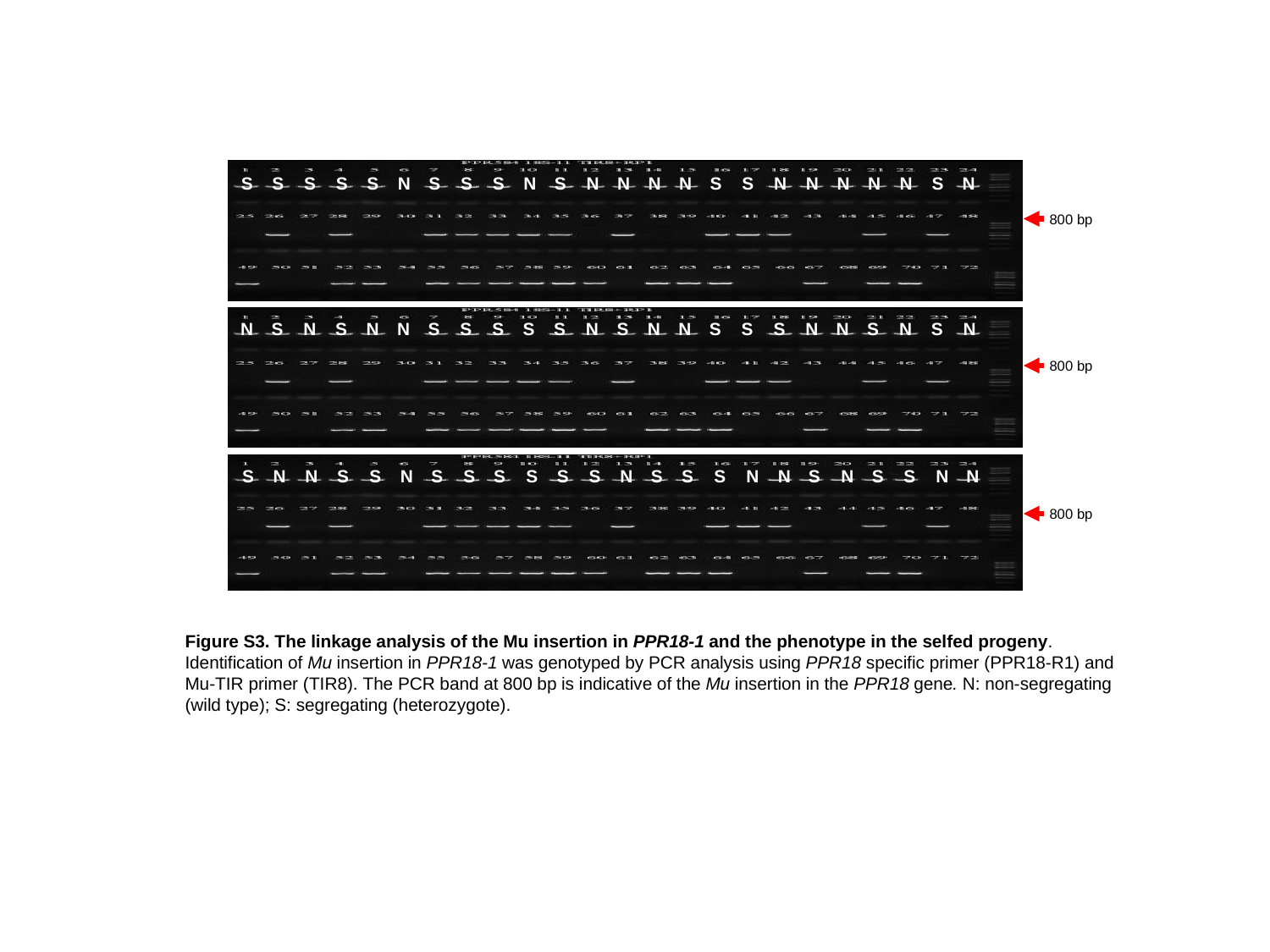

S
S
S
S
S
N
S
S
S
N
S
N
N
N
N
S
S
N
N
N
N
N
S
N
N
S
N
S
N
N
S
S
S
S
S
N
S
N
N
S
S
S
N
N
S
N
S
N
S
N
N
S
S
N
S
S
S
S
S
S
N
S
S
S
N
N
S
N
S
S
N
N
800 bp
800 bp
800 bp
Figure S3. The linkage analysis of the Mu insertion in PPR18-1 and the phenotype in the selfed progeny.
Identification of Mu insertion in PPR18-1 was genotyped by PCR analysis using PPR18 specific primer (PPR18-R1) and Mu-TIR primer (TIR8). The PCR band at 800 bp is indicative of the Mu insertion in the PPR18 gene. N: non-segregating (wild type); S: segregating (heterozygote).

## Slide 4
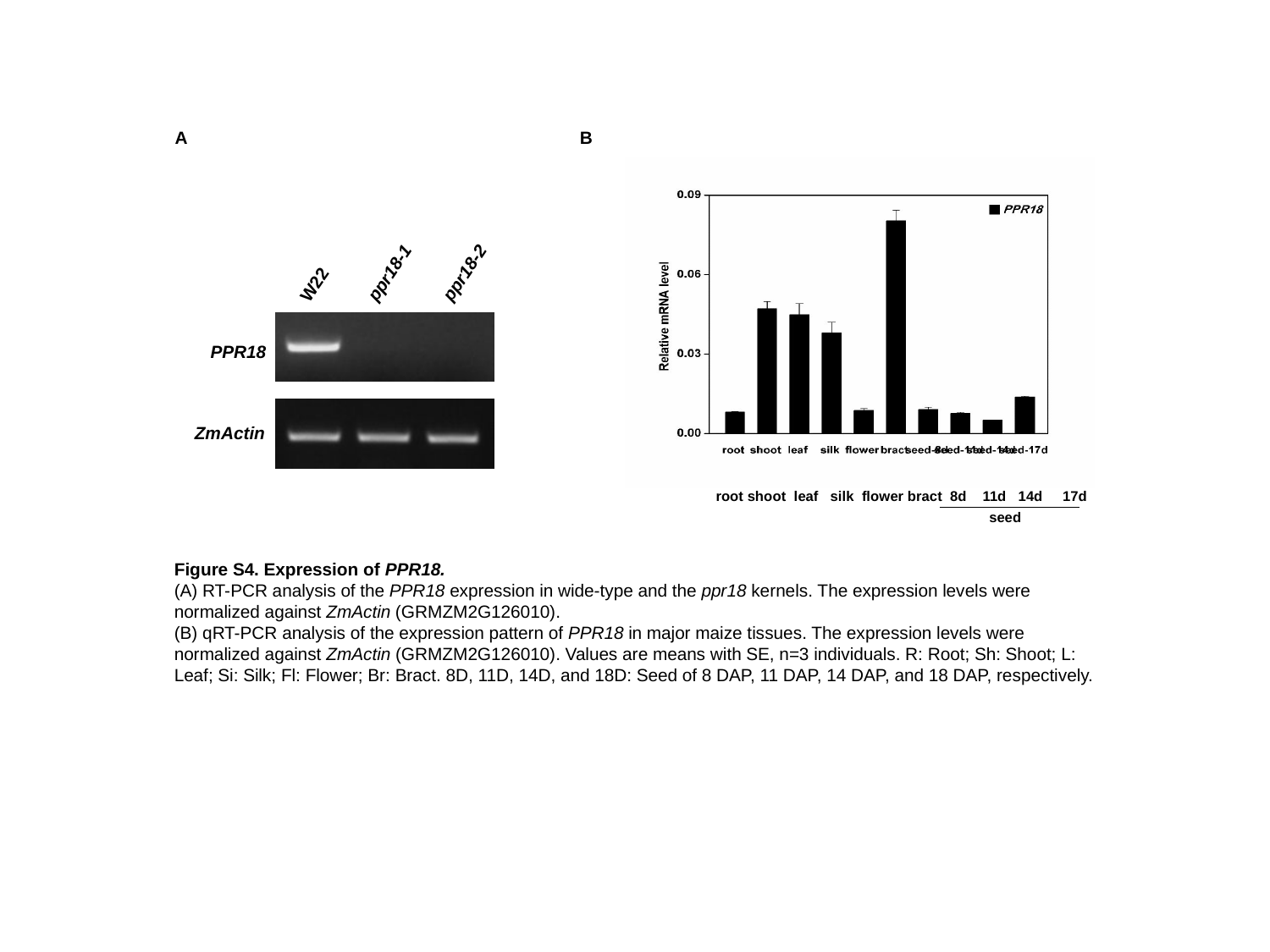

A
B
root shoot leaf silk flower bract 8d 11d 14d 17d
seed
ppr18-1
ppr18-2
W22
PPR18
ZmActin
Figure S4. Expression of PPR18.
(A) RT-PCR analysis of the PPR18 expression in wide-type and the ppr18 kernels. The expression levels were normalized against ZmActin (GRMZM2G126010).
(B) qRT-PCR analysis of the expression pattern of PPR18 in major maize tissues. The expression levels were normalized against ZmActin (GRMZM2G126010). Values are means with SE, n=3 individuals. R: Root; Sh: Shoot; L: Leaf; Si: Silk; Fl: Flower; Br: Bract. 8D, 11D, 14D, and 18D: Seed of 8 DAP, 11 DAP, 14 DAP, and 18 DAP, respectively.

## Slide 5
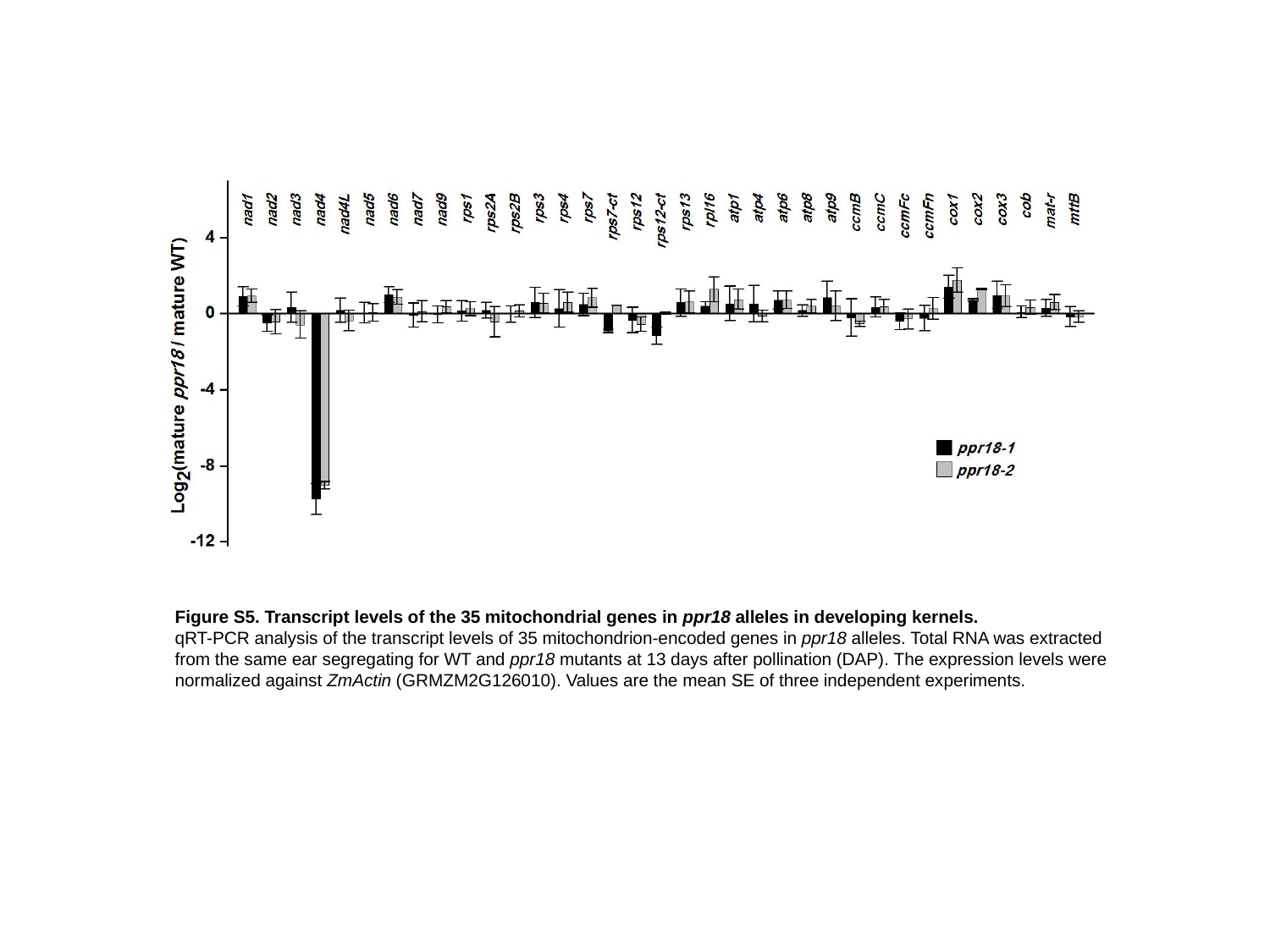

## Slide 6
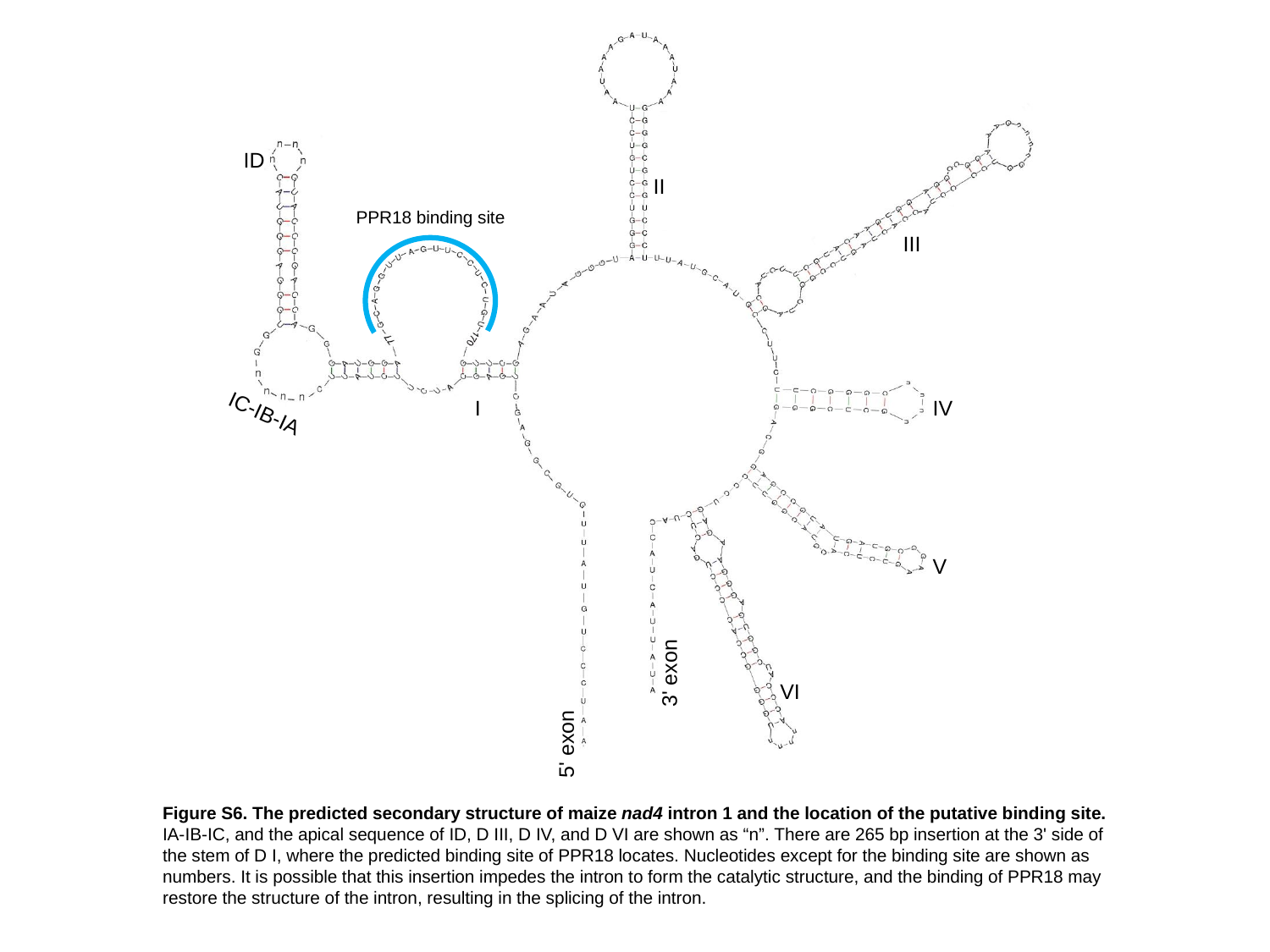

ID
II
PPR18 binding site
III
I
IV
IC-IB-IA
V
3' exon
VI
5' exon
Figure S6. The predicted secondary structure of maize nad4 intron 1 and the location of the putative binding site.
IA-IB-IC, and the apical sequence of ID, D III, D IV, and D VI are shown as “n”. There are 265 bp insertion at the 3' side of the stem of D I, where the predicted binding site of PPR18 locates. Nucleotides except for the binding site are shown as numbers. It is possible that this insertion impedes the intron to form the catalytic structure, and the binding of PPR18 may restore the structure of the intron, resulting in the splicing of the intron.

## Slide 7
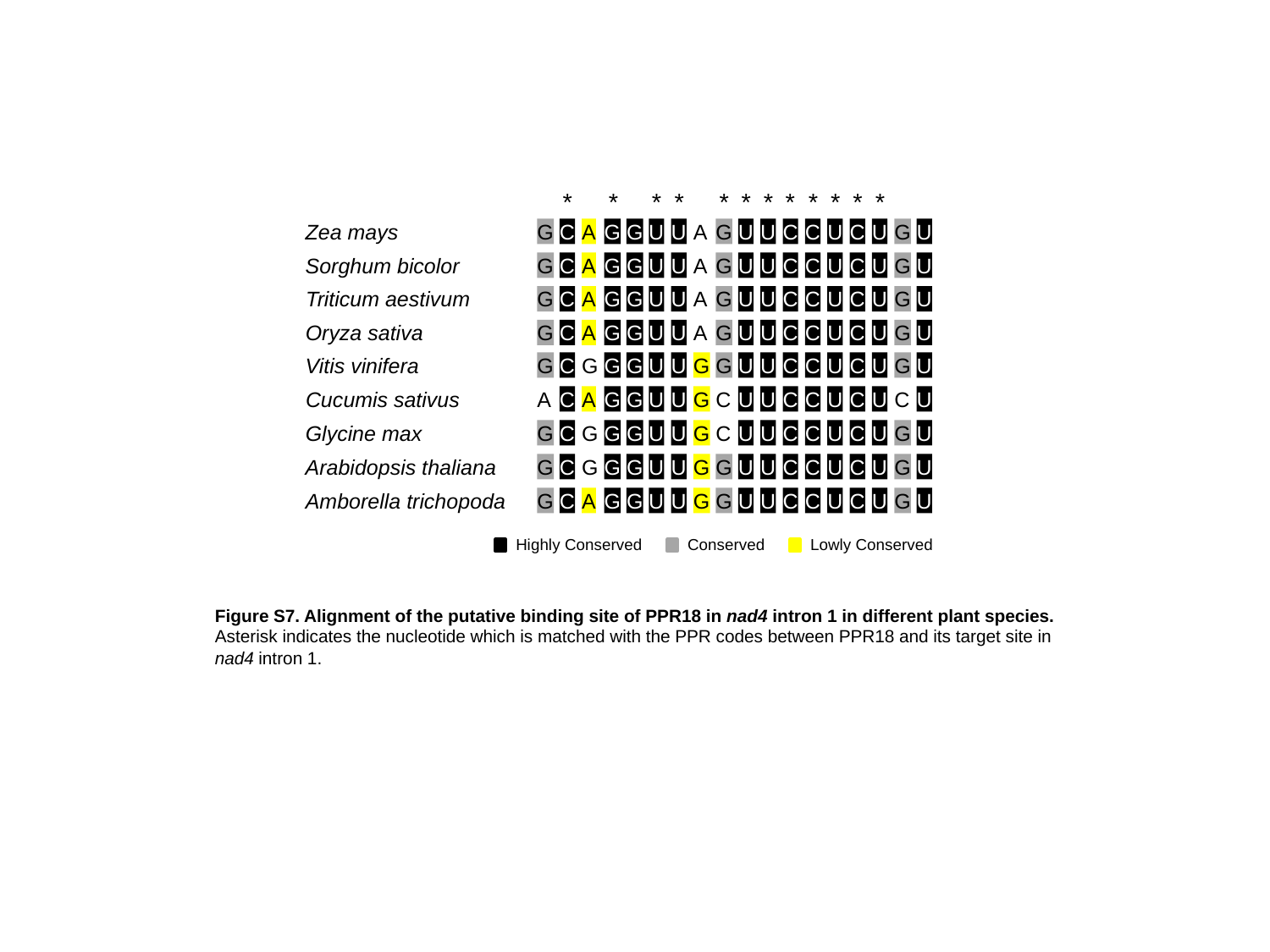

*
*
*
*
*
*
*
*
*
*
*
*
G
C
A
G
G
U
U
A
G
U
U
C
C
U
C
U
G
U
G
C
A
G
G
U
U
A
G
U
U
C
C
U
C
U
G
U
G
C
A
G
G
U
U
A
G
U
U
C
C
U
C
U
G
U
G
C
A
G
G
U
U
A
G
U
U
C
C
U
C
U
G
U
G
C
G
G
G
U
U
G
G
U
U
C
C
U
C
U
G
U
A
C
A
G
G
U
U
G
C
U
U
C
C
U
C
U
C
U
G
C
G
G
G
U
U
G
C
U
U
C
C
U
C
U
G
U
G
C
G
G
G
U
U
G
G
U
U
C
C
U
C
U
G
U
G
C
A
G
G
U
U
G
G
U
U
C
C
U
C
U
G
U
Zea mays
Sorghum bicolor
Triticum aestivum
Oryza sativa
Vitis vinifera
Cucumis sativus
Glycine max
Arabidopsis thaliana
Amborella trichopoda
Highly Conserved
Conserved
Lowly Conserved
Figure S7. Alignment of the putative binding site of PPR18 in nad4 intron 1 in different plant species.
Asterisk indicates the nucleotide which is matched with the PPR codes between PPR18 and its target site in nad4 intron 1.

## Slide 8
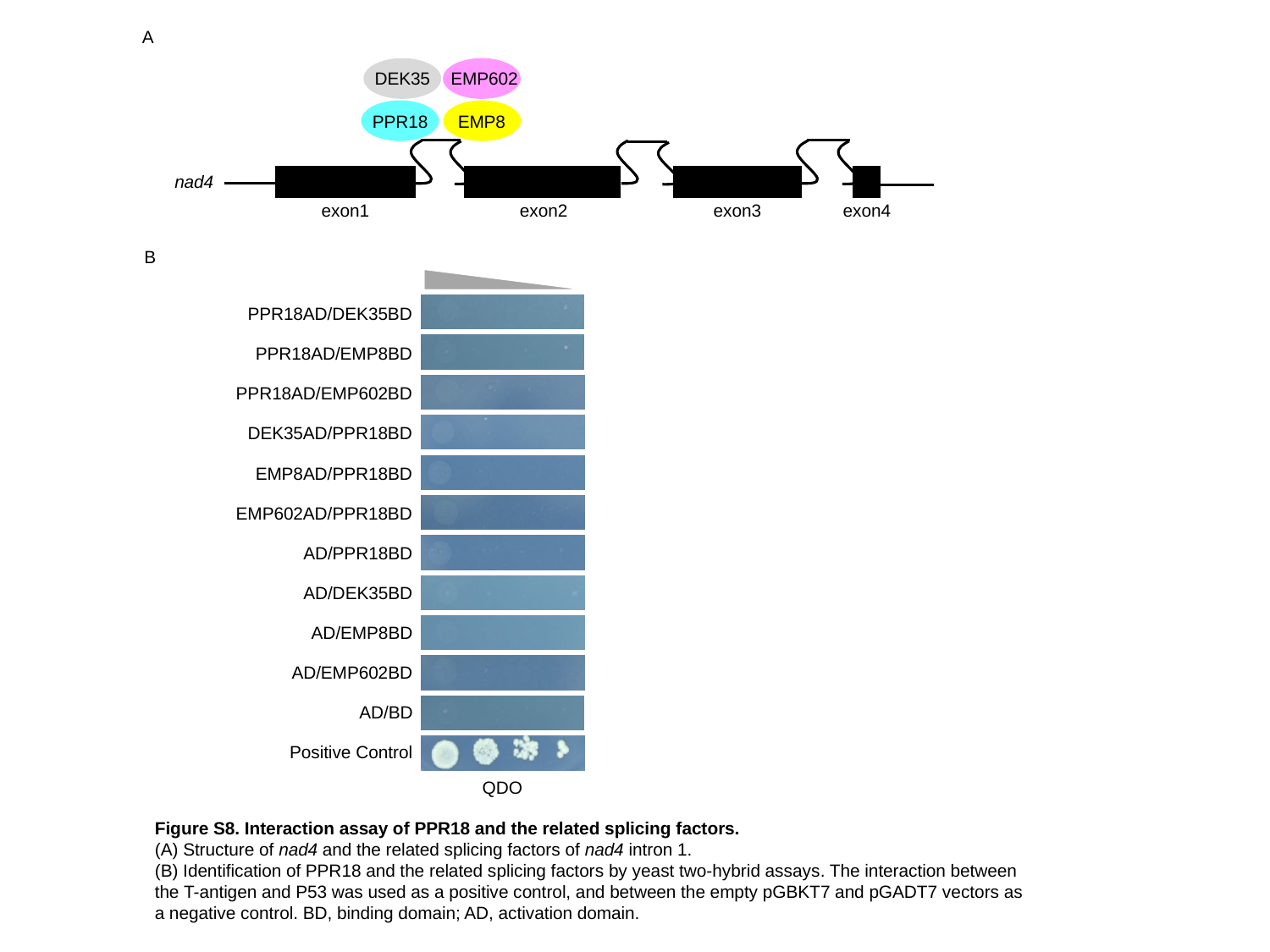

A
DEK35
EMP602
PPR18
EMP8
nad4
exon1
exon2
exon3
exon4
B
PPR18AD/DEK35BD
PPR18AD/EMP8BD
PPR18AD/EMP602BD
DEK35AD/PPR18BD
EMP8AD/PPR18BD
EMP602AD/PPR18BD
AD/PPR18BD
AD/DEK35BD
AD/EMP8BD
AD/EMP602BD
AD/BD
Positive Control
QDO
Figure S8. Interaction assay of PPR18 and the related splicing factors.
 Structure of nad4 and the related splicing factors of nad4 intron 1.
(B) Identification of PPR18 and the related splicing factors by yeast two-hybrid assays. The interaction between the T-antigen and P53 was used as a positive control, and between the empty pGBKT7 and pGADT7 vectors as a negative control. BD, binding domain; AD, activation domain.
